# High-grade neuroendocrine small-cell carcinoma of the anal canal: Long-term remission with chemoradiotherapy

**Published:** 2021-02-02

**Authors:** Roy Hajjar, Carole S. Richard, Francine Aubin, Marie-Pierre Campeau, Geneviève Soucy, Éric De Broux

**Affiliations:** ^1^Digestive Surgery Service, Centre hospitalier de l’Université de Montréal, Montréal, Québec, Canada; ^2^Hematology Oncology Service, Centre hospitalier de l’Université de Montréal, Montréal, Québec, Canada; ^3^Department of Radiation Oncology, Centre hospitalier de l’Université de Montréal, Montréal, Québec, Canada; ^4^Department of Pathology, Centre hospitalier de l’Université de Montréal, Montréal, Québec, Canada

**Keywords:** neuroendocrine small-cell carcinoma, anal canal, chemoradiotherapy

## Abstract

Primary small-cell carcinoma of the anal canal is an exceedingly rare tumor with a poor prognosis even when aggressive therapy is initiated. We present the case of a 53-year-old male patient who presented with chronic anal pain. Examination under general anesthesia revealed the presence of a mass in the anal canal. A biopsy was performed, and histopathological examination showed a high-grade neuroendocrine small-cell carcinoma. Assessment with endoscopic ultrasound showed an invasion of the internal anal sphincter. The patient was treated with a chemoradiotherapy (CRT) regimen consisting of cisplatin and etoposide, combined to radiotherapy. The patient achieved long-term remission with CRT. This is one of the first reports in the literature of a case of a high-grade neuroendocrine small-cell carcinoma of the anal canal where long-term remission was achieved with non-surgical management of a tumor invading the anal sphincter. This favorable evolution with CRT suggests that remission could still be achieved with anal small-cell carcinomas. More cases are however required to validate this approach.

**Relevance for Patients:**

This case presentation suggests that long-term remission can still be achieved using CRT and without an extensive surgical resection in patients with small-cell carcinoma of the anal canal.

## 1. Introduction

Neoplasms of the anal canal are rare and account for <5% of the malignancies of the gastrointestinal tract [1]. The majority of anal cancers are squamous cell carcinomas and adenocarcinomas, with prognosis and treatments that are well-defined [2]. They are followed less commonly by melanomas, basaloid carcinomas, leiomyosarcomas, and small cell carcinomas [3].

Neuroendocrine small cell carcinomas of the anal canal are exceedingly rare, with very few previously reported cases in the medical literature. They have an aggressive behavior and are commonly associated with poor outcomes [4,5]. Their best treatment remains uncertain as most of the current knowledge about their evolution and prognosis is mainly derived from case reports and small case series. Nearly all reported cases are associated with lethal recurrences despite aggressive medical and surgical management.

To the best of our knowledge, no previous cases of long-term remission and survival have been reported with non-surgical management of anal neuroendocrine small cell carcinomas.

## 2. Case Presentation

A 53-year-old male patient was evaluated at our institution for anal pain. His past medical history is unremarkable except for tobacco use and previous hemorrhoidectomies. The patient had undergone a colonoscopy in the previous year that was normal. At that moment, the anal canal was reported as irregular and biopsies were performed and displayed benign superficial hyperplastic changes.

**Figure 1:**
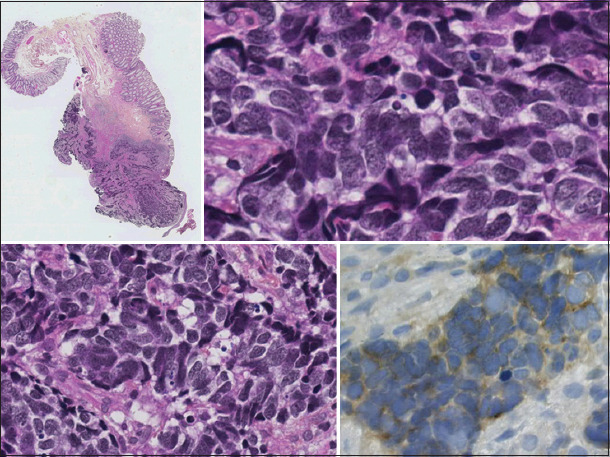
Histopathological analysis of the biopsy specimen depicting hematoxylin and eosin staining of the tumor tissue (upper right and lower left, ×40) and synaptophysin staining (lower right, ×40).

Due to chronic and refractory anal pain, the decision was to perform an anorectal examination under general anesthesia, which showed an anterolateral ulcerated lesion suggestive of a neoplasm. Biopsies were performed. Histopathological analysis revealed the presence of a high-grade neuroendocrine small-cell carcinoma [Figure 1]. The proliferation was located at the squamocolumnar junction of the anal canal. The biopsy showed a submucosal proliferation of malignant cells with a high nucleo/cytoplasmic ratio and pleomorphism. A high number of apoptotic bodies and crush artefacts were observed. The malignant cells were shown to express keratins, synaptophysin and few residual cells expressed chromogranin B. Squamous, melanocytic, and lymphocytic markers were negative.

**Figure 2:**
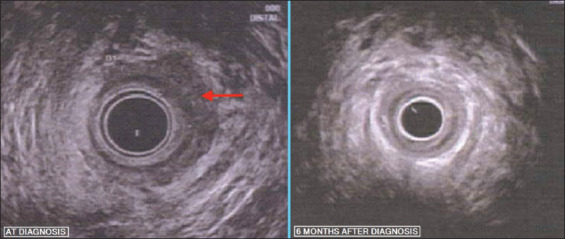
Endoscopic ultrasound at diagnosis and after chemoradiotherapy. The red arrow points toward an anal tumor that was noted on the initial examination.

An endoscopic ultrasound (EUS) was performed and showed a 2 × 1 cm anorectal lesion invading the superior portion of the internal anal sphincter [Figure 2]. No suspicious lymph nodes were noted. A positron emission tomography scan was performed and showed a hypermetabolic lesion in the anal canal with a maximum standardized uptake value (SUVmax) of 4,1. A computed tomography (CT) scan of the abdomen and pelvis failed to show the primary lesion and did not display any metastases. Thoracic and cerebral CT scans did not show any metastatic lesions. A benign appearing pulmonary nodule was noted. An octreoscan was performed and did not show any suspicious lesions.

The case was discussed at the multidisciplinary cancer committee and the joint decision was to proceed with chemoradiotherapy (CRT). The patient received 4 cycles of cisplatin and etoposide during a period of 4 months. The first chemotherapy cycle was not well-tolerated, and the regimen was thus modified for the 3 remaining cycles [Table 1]. The second and third treatments were administered during concomitant radiotherapy treatments. The patient was treated using an intensity-modulated radiation therapy technique. The macroscopic disease was treated to a total of 54 grays (Gy) in 30 fractions; 45 Gy were delivered prophylactically to inguinal and pelvic lymph nodes. The patient still complained of significant anorectal pain that subsided slowly several months after the end of CRT.

**Table 1:** Chemotherapy regimen

	Cycle 1		Cycle 2		Cycle 3		Cycle 4	
Day 1	Cisplatin (IV)	60 mg/m^2^	Cisplatin (IV)	25 mg/m^2^	Cisplatin (IV)	25 mg/m^2^	Cisplatin (IV)	25 mg/m^2^
	Etoposide (IV)	120 mg/m^2^	Etoposide (IV)	100 mg/m^2^	Etoposide (IV)	100 mg/m^2^	Etoposide (IV)	100 mg/m^2^
Day 2	Etoposide (IV)	120 mg/m^2^	Cisplatin (IV)	25 mg/m^2^	Cisplatin (IV)	25 mg/m^2^	Cisplatin (IV)	25 mg/m^2^
			Etoposide (IV)	100 mg/m^2^	Etoposide (IV)	100 mg/m^2^	Etoposide (IV)	100 mg/m^2^
Day 3	Etoposide (IV)	120 mg/m^2^	Cisplatin (IV)	25 mg/m^2^	Cisplatin (IV)	25 mg/m^2^	Cisplatin (IV)	25 mg/m^2^
			Etoposide (IV)	100 mg/m^2^	Etoposide (IV)	100 mg/m^2^	Etoposide (IV)	100 mg/m^2^

IV: Intravenous

A CT scan of the thorax, abdomen, and pelvis was performed 6 months after the diagnosis and was unremarkable except for benign appearing pulmonary nodularities. A pelvic magnetic resonance imaging (MRI) was performed at the same moment and revealed a T2 hyperintense signal in the anal canal that involved the internal sphincter, which was suggestive of a residual tumor or a radiation reaction. A second EUS was thus performed and was normal with no objectified residual mass [Figure 2]. Anoscopy and digital rectal examination were further performed and showed a scar at the site of the lesion with no evidence of a residual tumor. A second MRI of the pelvis was performed 1 year after the diagnosis and was normal. During follow-up, total and left-sided colonoscopies were performed 24 and 38 months, respectively, after the diagnosis and did not show any residual cancer.

Clinical follow-up was carried at the outpatient colorectal surgery clinic every 3-4 months with a digital rectal examination and anoscopy up to 30 months after the diagnosis. The frequency of outpatient visits was subsequently modified to once every 6 months. A total colonoscopy was performed 78 months after the diagnosis and showed a scar at the site of the lesion with no evidence of tumor recurrence. It is worth noting that thoracic scans were performed at 12, 19, and 26 months after the diagnosis to follow the benign appearing pulmonary nodules and were stable with no suspicious neoplastic lesions. The patient is disease-free 8 years after the initial diagnosis.

## 3. Discussion

We hereby present one of the first cases in the literature of a neuroendocrine small cell carcinoma of the anal canal with a complete long-term remission after a non-surgical CRT treatment.

Extra-pulmonary small cell carcinomas (EPSCC) are rare with a reported incidence of up to 0.4% [1,6]. The majority of these neoplasms occur mainly in the esophagus, colon, and rectum [1,6]. EPSCC of the anal canal is an exceedingly rare disease with very few previously reported cases. Table 2 summarizes the cases of small cell carcinomas of the anal canal that was detailed in the literature in the form of case reports or case series.

**Table 2:** Detailed cases of small cell carcinoma of the anal canal in the literature

Authors	Year	N	Age	Sex	Disease extent at diagnosis	Management	Evolution
Boman *et al*. [7]	1984	5	NA	NA	Distant metastasis	NA	Death after a median period of 2.2 months.
		1			Local (no sphincter invasion)	APR	Survival >5 years without recurrence.
		1			Local (external sphincter invasion)		Recurrence after a median period of 4 months. Death after a median period of 6 months after the surgery.
		5			Regional lymph nodes involved		
		1			NA	RT	Death 8.3 months after the diagnosis.
Nakahara *et al*. [9]	1993	1	48	Male	Local	RT	Pelvic recurrence with enlarged lymph nodes around the right common iliac vessels and in the left obturator cavity (treated with APR, lymph node dissection, and resection of the left internal iliac vessels). Recurrence (paraaortic lymph nodes, and lung metastases) 6 weeks after the surgery (treated with Cisplatin and Etoposide). Death due to suicide 11 weeks after the surgery.
Chapet *et al*. [2]	2001	2	30	Female	Local	CRT (with Cisplatin and 5-FU)	Recurrence 6 months after the diagnosis with hepatic metastases. Death with palliative CT.
			51	Female	Internal sphincter and perirectal fat invasion. 6 mm perirectal lymph node noted on EUS	APR (with inferior mesenteric and iliac lymph node dissection) and CRT (with Vepesid and Cisplatin). Second line CT: Adriamycin. Third line CT: 5-FU	Pulmonary metastases 17 months after the diagnosis. Death 30 months after the diagnosis.
Kobayashi *et al*. [19]	2006	1	63	Female	Local	APR	Good evolution at 6 months of follow-up
Meyer *et al*. [20]	2007	1	47	Female	Hepatic and pulmonary metastases	CRT (with Cisplatin and Etoposide)	Death 10 months after the diagnosis.
Alcindor *et al*. [10]	2008	1	45	Male	Metastases to the liver and to abdominopelvic lymph nodes	CRT (with Cisplatin and Etoposide)	Death 6 months after the diagnosis.
Doddi *et al*. [3]	2009	1	60	Female	Local	CRT (with Cisplatin and Etoposide)	Distant recurrence with liver and lung metastasis (treated with palliative CT). Death 18 months after the diagnosis.
Khan *et al*. [16]	2009	1	50	Female	Locally invasive 3.8×3.6 cm mass with borderline enlarged perirectal lymph nodes	CRT (with Cisplatin and Etoposide)	Local presacral recurrence after 2 years (treated with surgical resection then Cisplatin and Irinotecan). Bone metastases 2 years later (treated with Zometa and Topotecan at last reported follow-up).
Cimino-Mathews *et al*. [21]	2012	5	45-62	Female	NA	NA	Median survival: 18.7 months (range: 13-22)*
Ebehardt *et al*. [1]	2012	1	63	Female	Metastases to a right inguinal lymph node	CRT (with Cisplatin and Etoposide)	Metastases (to the pancreas, adrenal gland, liver, breast, lung, brain, and lymph nodes) 3 months after CRT. Death 10 months after the diagnosis.
Ohtomo *et al*. [11]	2012	1	70	Female	Local	APR	Pulmonary and bone metastases 1 year after the surgery. Death 2 years after the surgery with palliative RT.
Borgonovi Christiano *et al*. [22]	2012	1	49	Female	Local	Nigro CRT protocol (initial diagnosis was that of a squamous cell carcinoma	Metastases in distant lymph nodes and in the greater omentum, with septic complications including inguinal lymph nodes abscesses and Fournier syndrome. Death 21 months after initial diagnosis
Marcus *et al*. [12]	2013	1	49	Male	Metastasis in perirectal and inguinal lymph nodes on PET-scan	CRT (with Cisplatin and Etoposide)	No evidence of residual tumor with a sigmoidoscopy 5 months after CRT.
Ghahramani *et al*. [23]	2014	1	50	Male	Liver metastasis	CT and diverting colostomy	Death 12 days after the operation.
Khmou *et al*. [6]	2014	1	53	Male	Local	CRT	Death due to metastatic disease 12 months after diagnosis.
Lee *et al*. [24]	2015	1	56	Male	Enlarged left inguinal lymph node, liver metastasis, and a possible lung metastasis	CRT (with Carboplatin and Etoposide)	Death after 1 cycle of CT.
Surag *et al*. [8]	2016	1	46	Male	Enlarged inguinal, mesorectal and internal iliac lymph nodes on CT scan	APR+CT	Death after 4 months with liver metastases.
Gates *et al*. [25]	2020	1	68	Female	Local mass in the anal canal	Chemoradiotherapy	NA
Juhlin *et al*. [4]	2020	1	37	Male	Local	APR with adjuvant CRT (cisplatin and etoposide)	Recurrences in the inguinal lymph nodes and abdominal wall, managed with surgical excision and CT (no recurrences 13 years after initial presentation)

CRT: Chemoradiotherapy; 5-FU: 5-Fluorouracil; CT: Chemotherapy; EUS: Endoscopic ultrasound; APR: Abdominoperineal resection; NA: Not available; RT: Radiotherapy; PET: Positron emission tomography.

* Data for 3 patients

It is worth noting that the majority of cases describe a disease with an aggressive phenotype even when an initial response to surgical or non-surgical treatment is present. Only one previous case, reported by Boman *et al*. in 1983, documents a long-term (>5 years) disease-free survival in a patient who underwent an abdominoperineal resection (APR) [7]. It is important to note however that the patient had a localized disease with no sphincter invasion at the time of diagnosis. No previous detailed cases were found to describe a long-term disease-free survival with the sole use of CRT.

The diagnosis of small-cell carcinoma in the anal canal relies in reported cases on neuroendocrine differentiation [1,3,8]. Neuroendocrine markers that have been described in such neoplasms include synaptophysin, CD56, CD57, and thyroid transcription factor (TTF-1) [3,8]. TTF-1 is commonly expressed in small cell lung carcinoma and might be helpful in differentiating metastasis from a primary anal tumor [3].

On another note, a question has already been raised over the potential relation of anal small-cell carcinomas and the human immunodeficiency virus (HIV). Several previous reports have described anal EPSCC in patients with HIV, questioning the potential immunosuppressive effect of HIV on the development of this rare tumor [9,10]. Ohtomo *et al*. have in addition raised in 2012 the hypothesis of a potential relation with the human papillomavirus 18 (HPV-18), which is a well-established oncogenic factor in the tumorigenesis of anal squamous-cell carcinoma [11]. Marcus *et al*. have even suggested in 2013 that anal EPSCC may arise from squamous dysplasia and carcinoma *in situ* [12]. Mutated genes involved in the carcinogenesis of the more common squamous cell carcinoma include the PIK3CA, MLL2, and MLL3 genes [13,14]. Activating mutations in the PIK3CA gene may thus be a potential therapeutic target in the management of anal cancer, namely squamous cell carcinoma in which PIK3CA mutations and HPV oncogenes promote carcinogenesis [15]. The role of these factors in anal EPSCC has not however been clearly elucidated and requires further investigation.

Due to the scarcity of anal EPSCC, no formal evidence-based recommendations have been issued with regard to its gold-standard management. It has been suggested that these tumors are equivalent to pulmonary small-cell carcinomas and can be thus treated is a similar manner [3,16]. Takada *et al*. have reported a 5-year survival rate of 23.7% in patients with limited-stage small-cell lung cancer who received four cycles of cisplatin and etoposide with concurrent radiotherapy [17]. As mentioned by Doddi *et al.*, the mainstay of treatment may be a cisplatin-based chemotherapy with etoposide [3]. Radiotherapy is an adjunct that helps control locoregional disease [8]. Chemotherapy is used as a radiosensitizer to increase the efficacy of radiation therapy. It also aims at eradicating micrometastases. Hoskins *et al*. have shown in 2003 that small-cell carcinoma of the cervix can be successfully treated with a combination of a platinum-based chemotherapy and radiotherapy in 55% of patients [18]. CRT failed however to show such a significant curative response with anal EPSCC.

The role of surgical resection in the treatment of anal EPSCC remains uncertain since it does not seem to lead to long-term remission. Brieau *et al*. (2015) compared the outcomes of patients with non-metastatic anorectal neuroendocrine carcinoma subjected to a surgical treatment or to chemotherapy with or without radiotherapy [5]. No differences were noted among groups with progression-free survival of 13.0 and 13.2 months, respectively [5]. Surag *et al*. have suggested in 2016 that APR may be an option that does not have an impact on disease progression [8]. Furthermore, the review of previously reported cases shows that radical surgical excision, including APR with or without lymphadenectomy, did not prevent relatively fast recurrences in almost all of the cases where it has been used. This is not without pointing out that APR is a major procedure with a permanent colostomy and significant associated morbidity.

Our patient responded well to upfront CRT with cisplatin/etoposide and maintained an adequate sphincter tone. Local pelvic pain has been described in several previous reports as a potential sign of neoplastic relapse. The presence of chronic persistent pain in our patient might have complicated clinical follow-up as clinical symptoms could not be used as predictors of local recurrence. Nonetheless, despite the presence of local pelvic pain that took several months to subside, the patient did not display any signs of recurrence and is still deemed disease-free 8 years after the diagnosis. According to previous data, this may be one of the first reports that describe a favorable long-term outcome with an anal EPSCC involving the anal sphincter and subjected only to CRT. With past cases reporting bad outcomes in the majority of non-surgically managed patients, it is tempting to opt for radical resection as a curative approach when the disease is still localized. Surgery failed however to show its superiority over CRT in the few past recorded cases.

## 4. Conclusion

Neuroendocrine small-cell carcinomas of the anal canal are exceedingly rare tumors with very few previously described cases. Prognosis and survival are generally unfavorable even when the disease is initially localized and aggressive therapeutic modalities are used. A regimen of cisplatin and etoposide combined to local radiotherapy induced a complete long-term remission in our patient who initially had an invasion of the internal sphincter. This favorable evolution suggests that a complete long-term recovery could still be achieved with this disease. The specific factors that may have contributed to the favorable evolution of the disease in this case remain unknown. One might speculate that the localized non-metastatic disease contributed to the good response to treatment. More cases are however required to determine prognostic factors, to validate treatment modalities, and to determine the best management approach.

### Conflicts of Interest

The authors declare that they have no conflicts of interest.

### Informed Consent

Informed consent was obtained from the patient for the publication of the present manuscript.

### Funding

None.
